# Predator Avoidance in Extremophile Fish

**DOI:** 10.3390/life3010161

**Published:** 2013-02-06

**Authors:** David Bierbach, Matthias Schulte, Nina Herrmann, Claudia Zimmer, Lenin Arias-Rodriguez, Jeane Rimber Indy, Rüdiger Riesch, Martin Plath

**Affiliations:** 1 Department of Ecology & Evolution, J.W. Goethe University Frankfurt, Max-von-Laue-Straße 13, Frankfurt am Main, D-60438, Germany; E-Mails: cla.zim.uni@gmail.com (C.Z.); mplath@bio.uni-frankfurt.de (M.P.); 2 Institute of Biochemistry & Biology, Unit of Animal Ecology, University of Potsdam, Maulbeerallee 1, Potsdam, 14469, Germany; E-Mails: schnulli83@gmx.de (M.S.); nina-caterina@web.de (N.H.); 3 División Académica de Ciencias Biológicas, Universidad Juárez Autónoma de Tabasco (UJAT), Villahermosa, Tabasco, CP 86150, México; E-Mails: leninariasrodriguez@hotmail.com (L.A.-R); jeanerimberindy@yahoo.com (J.R.I.); 4 Department of Animal and Plant Sciences, University of Sheffield, Western Bank, Sheffield S10 2TN, UK; E-Mail: rwriesch.evolutionarybiology@gmail.com

**Keywords:** antipredator behavior, hydrogen sulfide, *Poecilia*, predator avoidance, predator recognition

## Abstract

Extreme habitats are often characterized by reduced predation pressures, thus representing refuges for the inhabiting species. The present study was designed to investigate predator avoidance of extremophile populations of *Poecilia mexicana* and *P. sulphuraria* that either live in hydrogen sulfide-rich (sulfidic) springs or cave habitats, both of which are known to have impoverished piscine predator regimes. Focal fishes that inhabited sulfidic springs showed slightly weaker avoidance reactions when presented with several naturally occurring predatory cichlids, but strongest differences to populations from non-sulfidic habitats were found in a decreased shoaling tendency with non-predatory swordtail (*Xiphophorus hellerii*) females. When comparing avoidance reactions between *P. mexicana* from a sulfidic cave (Cueva del Azufre) and the adjacent sulfidic surface creek (El Azufre), we found only slight differences in predator avoidance, but surface fish reacted much more strongly to the non-predatory cichlid *Vieja bifasciata*. Our third experiment was designed to disentangle learned from innate effects of predator recognition. We compared laboratory-reared (*i.e.*, predator-naïve) and wild-caught (*i.e.*, predator-experienced) individuals of *P. mexicana* from a non-sulfidic river and found no differences in their reaction towards the presented predators. Overall, our results indicate (1) that predator avoidance is still functional in extremophile *Poecilia* spp. and (2) that predator recognition and avoidance reactions have a strong genetic basis.

## 1. Introduction

Falling victim to predation excludes an individual from future reproductive opportunities, thus underpinning the importance of appropriate anti-predator behavior to prevent predator-related mortalities [1]. Before a prey species can react to a predator, however, initial recognition is required either through visual [2,3,4], olfactory [5], tactile [6,7], or auditory cues [8]. Upon this initial detection, the prey then has to assess the likelihood of an attack, which is crucial for triggering an appropriate avoidance response [2]. For the purpose of the present study, we define “predator avoidance” as both detection and identification of predatory stimuli (here: visual cues from piscivorous cichlids) that elicit an avoidance response in prey (*Poecilia mexicana*, a small, neotropical livebearing fish).

Anti-predator behavior is typically associated with some kind of cost [9], and populations are predicted to rapidly loose these expensive behaviors when colonizing low-predation or predator-free environments, in which the costs outweigh potential benefits [10,11]. Economic considerations dictate that this reduction process should be even faster in habitats with low resource availability. Nonetheless, some anti-predator behaviors are known to persist throughout generations following initial isolation from predators [12,13,14]. On top of that, several studies exemplified that anti-predator responses in natural populations represent a combination of both innate and experiential (*i.e.*, learned) components [15,16,17,18]. Learned responses are lost after one generation, and thus need to be reacquired in every generation [17], while genetically based behaviors may persist for many thousand generations, but loss may be permanent [15].

A unique opportunity to study the evolution of anti-predator response mechanisms is provided near the Southern Mexican cities of Teapa and Tapijulapa, which are located in the state of Tabasco. Here, *P. mexicana* inhabits various streams and rivers with diverse fish faunas including several piscivorous species [19,20]. Additionally, locally adapted populations in at least three different tributaries of the Río Grijalva drainage also inhabit springs characterized by the lack of piscivorous fishes in waters containing high amounts of hydrogen sulfide (H_2_S) [19,20]. H_2_S is acutely toxic to most metazoans even in micromolar amounts as it inhibits aerobic respiration, but also leads to extreme hypoxia in the water [21,22]. To cope with this toxicity and the H_2_S-induced hypoxia, locally adapted *P. mexicana* in sulfidic habitats resort to aquatic surface respiration (ASR), thus exploiting the more oxygen-rich air-water interface using their gills (e.g., [23,24]). However, ASR also exposes fish in sulfidic habitats to avian predation, which is up to twenty times higher than in non-sulfidic habitats [25]. Another feature of those extreme habitats is low energy availability [23]. While the predominant food source of non-sulfidic surface-dwelling *P. mexicana* is detritus and green algae, diets of conspecifics in the sulfidic surface and cave streams are dominated by chemoautotrophic (sulfur) bacteria and aquatic invertebrates [26]. Fish in the sulfidic habitats spend up to 85% of their time performing ASR, which strongly reduces the time afforded for feeding [23]. In combination with the energy demanding detoxification of H_2_S [22], this leads to lower general body conditions (weight-length ratios [19], abdominal distention [27], and body fat content [28,29]) observed in H_2_S-adapted *P. mexicana*. As a result, energy limitation along with reduced piscine predation rates should favor the rapid reduction of costly anti-predator behaviors in all extremophile *P. mexicana* populations.

**Figure 1:**
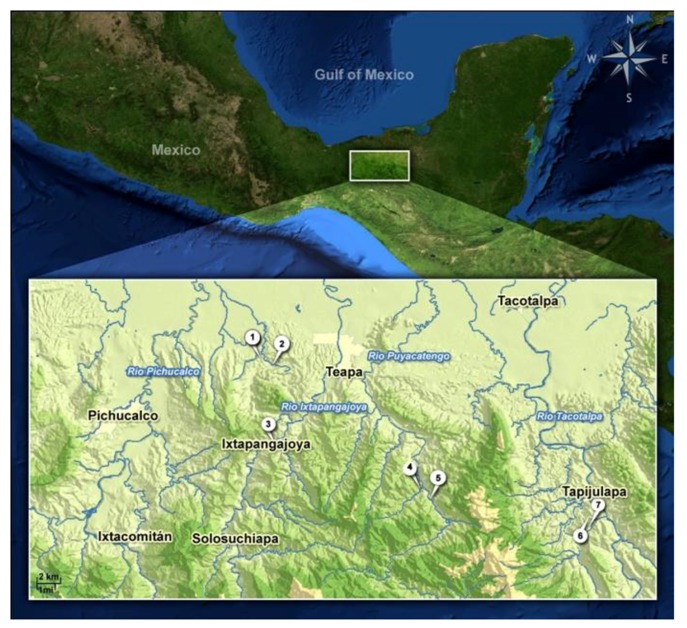
Overview of the study area and detailed view of the collection sites. (**1**) Baños del Azufre (sulfidic); (**2**) Río El Azufre (non-sulfidic); (**3**) Río Ixtapangjoya (non-sulfidic); **(4**) Río Puyacatengo (non-sulfidic); (**5**) La Lluvia (sulfidic); (**6**) Cueva del Azufre (cave, sulfidic); (**7**) El Azufre II (sulfidic).

In the present study, we investigated the time spent near or away from several predatory (and non-predatory) fishes as an estimate of anti-predator behavior in *Poecilia* spp. from three independent sulfur systems. In two of them, the Río Tacotalpa and Río Puyacatengo drainages, *P. mexicana* inhabits several sulfidic surface springs, but in the Río Tacotalpa drainage this species has also successfully colonized a sulfidic cave [30,31] (Figure 1). The sulfidic springs of the Baños del Azufre in the Río Pichucalco drainage, on the other hand, are inhabited by the sulfur molly (*Poecilia sulphuraria* [32]; Figure 1), a highly H_2_S-adapted sister species of *P. mexicana* [31].

Although the sulfur springs in these systems can be considered to be essentially free of predatory fish species, several small non-sulfidic streams drain into them, and predatory cichlids (e.g., *Cichlasoma salvini*), a predatory characid (*Astyanax aeneus*), as well as other poeciliids (e.g., *Heterandria bimaculata* and *Xiphophorus hellerii*) can be observed in the less toxic mixing zones [18,19,20]. The Cueva del Azufre, by contrast, is completely devoid of fish species other than *P. mexicana*, the sole exception being occasional sightings of the synbranchid eel *Ophisternon aenigmaticum *[20]. Nevertheless, *P. mexicana* inhabiting this cave face high predation pressure through a giant water bug, *Belostoma* sp. [33,34], several pisaurid, ctenid and theraphosid spiders [35], one species of trichodactylid freshwater crab, *Avotrichodactylus bidens *[36], possibly also an as yet undetermined pseudothelphusid freshwater crab (M.P., unpublished data), and most likely terrestrial mammals that regularly venture into the cave (based on mammal scat regularly found in different parts of the cave; all authors, personal observation).

We tested for differences in anti-predator behavior of *Poecilia *spp. from several extreme and benign habitats and thus analyzed avoidance reactions elicited by predatory fish species that naturally co-occur with *P. mexicana* in non-sulfidic sites [*C. salvini* and *Petenia splendida* (Cichlidae)]. We further included non-piscivorous species [green swordtails, *X. hellerii* (Poeciliidae) and *Vieja bifasciata *(Cichlidae)] that are also common in South Mexican sulfide-free habitats [37] in our experiments as stimuli for control treatments. We predicted that at least *X. hellerii* should not elicit an avoidance reaction [18] if focal fish were able to distinguish threatening and non-threatening stimuli.

Our first question was whether predator identification and subsequent avoidance were still functional in *Poecilia* spp. inhabiting sulfidic springs. We, therefore, compared predator avoidance reactions of populations from non-sulfidic habitats to those of populations inhabiting sulfidic springs in two of the three drainages. We predicted that fish from non-sulfidic waters would show strong avoidance reactions when confronted with the omnivorous *C. salvini *[37,38] and especially when confronted with the piscivorous *P. splendida *[37], while reactions of H_2_S-adapted *Poecilia* spp. would be less predictable.

In a second experiment, we looked for differences in the avoidance reactions towards the aforementioned predators between *P. mexicana* from the sulfidic Cueva del Azufre and the adjacent sulfidic surface creek, the El Azufre (Figure 1). Cave mollies are known to have acquired several adaptations related to their life in perpetual darkness: reduced shoaling [39] and reduced aggressive behavior [40,41,42]. However, while cave mollies have reduced eye size [43,44,45], their eyes do not differ in spectral sensitivity from those of their surface-dwelling counterparts, and light-reared fish can perceive and react to visual cues [46]. Both the sulfidic surface and the cave habitats are largely devoid of piscivorous fishes, but *P. mexicana* from the sulfidic El Azufre experience immense avian predation [25], and could at least occasionally come into contact with predatory fishes by entering the less sulfidic mixing zones that connect the El Azufre to the nearest non-sulfidic creeks [18,19,20]. *Poecilia mexicana* from the inside of the Cueva del Azufre, on the other hand, are highly unlikely to ever encounter piscivorous fishes or birds (see above). Thus, we predicted that the cave population—the sole example of a population that most likely evolved without any contact to predatory fishes—would show a reduction in predator avoidance reactions.

Any differences in predator avoidance found in our two previous experiments could probably either be the result of heritable differences between populations, namely, reductions of predator recognition and avoidance or, if predator recognition and avoidance are intact, of a lack of predator experience, leading to an altered evaluation of threat levels [2,18]. Our third experiment was, therefore, designed to disentangle learned from innate effects on predator avoidance reactions [2]. We used laboratory-reared (*i.e.*, predator-naïve) descendants of *P. mexicana* originated from the non-sulfidic Río Ixtapangajoya (Río Puyacatengo drainage; Figure 1) and compared their behavior to that of wild-caught (*i.e.*, predator-experienced) individuals from the same origin. Kelley and Magurran [4] found predator avoidance in the Trinidadian guppy (*Poecilia reticulata*) to be influenced by experience when comparing wild-caught and laboratory-reared (thus predator-naïve) fish. Ferrari *et al.* [47] could show that fathead minnows (*Pimephales promelas*) were able to learn to recognize predators when exposed to predator odors and even generalize their anti-predator response to new, closely-related predators. However, a recent study further revealed that predator-naïve *P. mexicana* females responded with a stronger change in mating preferences when exposed to a set of predators (and non-predators) than wild-caught, predator-experienced females [18]. The authors suggest innate predator recognition mechanisms that are fine-tuned by experience. Predator-experienced *P. mexicana* may be more capable to estimate the predator's motivation to prey—which was assumed to be low since live predators might have been stressed by the experimental handling [18]. Accordingly, we predicted predator-experienced individuals to show weaker predator avoidance responses to visual stimuli from live predatory cichlids than naïve ones.

## 2. Experimental Section

### 2.1. Study Organisms, Sampling Sites and Maintenance of the Test Animals

Poeciliid fishes are livebearers, and males use their transformed anal fin, the gonopodium, to transfer sperm [48]. Females store sperm to fertilize several consecutive, monthly broods, and sperm competition is intense [49]. The Atlantic molly (*Poecilia mexicana*) is widespread in various streams, lakes and lagoons along the Central American Atlantic coast [37]. While *P. mexicana* females have a cryptic body coloration, large males show conspicuous black vertical bars, and dominant males may even become completely black with yellowish to orange margins on the dorsal and anal fins [40]. Smaller males are typically less conspicuous in coloration. *Poecilia mexicana* males do not court females [50]; their pre-mating behavior consists of so-called genital nipping where males try to gather chemical information on female receptivity by making oral contact with the female's genital opening [40,50]. Nipping typically, but not always, precedes copulation and is the most frequent sexual behavior in *P. mexicana* [40].

Atlantic molly males usually establish dominance hierarchies, and dominant (typically the largest) males monopolize small shoals of females, which they aggressively defend against rivals [40]. For this study, *Poecilia* spp. were collected in sulfidic springs and adjacent non-sulfidic habitats in the Mexican states of Tabasco and Chiapas, particularly in the region around the city of Teapa. Here, the mountains of the Sierra Madre de Chiapas meet the wide floodplains of northern Tabasco. The six spring complexes known to be inhabited by *Poecilia* are located in the foothills of the Sierra Madre and distributed across three major tributaries of the Río Grijalva. In the upper reaches where the sulfidic springs are located, these tributaries (Ríos Tacotalpa, Puyacatengo, and Pichucalco) are separated by mountains, while they all eventually join the Río Grijalva and are widely interconnected in the lowlands at least during the wet season. In the Río Pichucalco drainage, the sulfidic ecotype has been described as a distinct species, *P. sulphuraria* (Alvarez 1948), which is endemic to sulfide spring complexes at the Baños del Azufre and Rancho La Gloria and represents a more ancient lineage of sulfide spring fish [31]. In the Tacotalpa and Puyacatengo drainages, H_2_S-adapted ecotypes cluster phylogenetically within Southern Mexican *P. mexicana*. Nonetheless, all three lineages share a series of traits characteristic of sulfidic spring fish [31]. In the Río Tacotalpa drainage, *P. mexicana* also colonize a sulfidic cave, the Cueva del Azufre [30,51]. The Cueva del Azufre is divided into 13 different chambers, with Chamber XIII being the innermost chamber (after [30]). Several springs in the cave (mainly in Chamber X) release sulfidic water, and the creek that flows through the cave eventually leaves the cave and turns into the sulfidic El Azufre.

For Experiments 1 and 2, we used wild-caught fish from different sulfidic and non-sulfidic surface habitats as well as fish from Chamber II of the Cueva del Azufre [19,30,31] (Table 1). Light enters the front parts of that chamber through several holes in the ceiling [45], so fish could be tested for a visual response under light conditions (even though the skylights are not sufficient to illuminate the chamber fully). Upon capture, fish were transferred into closed and aerated 38 L (43 × 31 × 32 cm) black Sterilite^®^ containers and brought immediately to the Tropical Aquaculture Laboratory at the División Académica de Ciencias Biológicas from the Universidad Juárez Autónoma de Tabasco (UJAT) in Villahermosa, Tabasco, Mexico (DACBIOL-UJAT). Here, they were kept separated by sex in well aerated 70 L tanks (filled with aged tap water) for 24 hours at 30.0 ± 1.0 °C, with approximately 12:12 hours light:dark cycle and could acclimate to the water conditions in the laboratory.

For Experiment 3, we used descendants of wild-caught fish of the second to fourth laboratory generation originating from the Río Ixtapangajoya (Table 1). Fish were reared in 1000-L tanks at DACBIOL-UJAT under semi-natural conditions, in absence of predators. In the laboratory all fish were fed once a day *ad libitum* with commercially available pellet and flake food. Experiments were conducted between 15 April and 15 July 2011.

**Table 1:** Standard length (SL ± S.E.M.), sample size (*N*, number of individuals) and GPS data for the eight populations studied.

Population	Species	Habitat / Treatment	SL [mm]		Sampling point	
			males	females	Latitude	Longitude
Río Ixtapangajoya (Río Puyacatengo drainage)	*Poecilia mexicana*	wc*, n, sf	28.4 ± 0.4 (*N *= 28)	28.9 ± 0.9 (*N *= 24)	17.49450	−92.99763
Río Ixtapangajoya (Río Puyacatengo drainage)	*P. mexicana*	lab, n, sf	39.5 ± 1.2 (*N *= 28)	43.67 ± 2.0 (*N *= 24)	17.49450	−92.99763
Río El Azufre (Río Pichucalco dranage)	*P. mexicana*	wc, n, sf	40.5 ± 2.2 (*N *= 28)	43.8 ± 1.6 (*N *= 24)	17.55634	−93.00762
Baños del Azufre (Río Pichucalco drainage)	*Poecilia sulphuraria*	wc, s, sf	24.6 ± 0.6 (*N *= 28)	26.04 ± 1.1 (*N *= 24)	17.55225	−92.99859
RíoPuyacatengo	*P. mexicana*	wc, n, sf	39.2 ± 1.3 (*N *= 28)	40.4 ± 2.3 (*N *= 25)	17.47000	−92.89573
La Lluvia (Río Puyacatengo drainage)	*P. mexicana*	wc, s, sf	28.2 ± 0.5 (*N *= 28)	35.8 ± 0.9 (*N *= 25)	17.46387	−92.89541
Cueva del Azufre (Río Tacotalpa drainage)	*P. mexicana*	wc, s, sf	31.1 ± 0.6 (*N *= 28)	39.3 ± 1.2 (*N *= 24)	17.44225	−92.77447
El Azufre II (Río Tacotalpa drainage)	*P. mexicana*	wc, s, ca	33.3 ± 0.9 (*N *= 28)	35.8 ± 1.1 (*N *= 24)	17.43843	−92.77476

*Habitat/treatment variables are defined as follows: wc, wild-caught; lab, laboratory-reared; n, non-sulfidic; s, sulfidic; sf, surface; ca, cave.

### 2.2. Experimental Design

Tests were conducted in two identical test tanks (42.6 × 30.0 × 16.5 cm) made of UV-transparent plexiglas. Each tank was visually divided into three equally-sized zones by black marks on the outside. The central zone was designated the neutral zone, the two lateral zones as preference zones. Predators were presented in one of two small auxiliary tanks (19.5 × 30.0 × 14.5 cm) on either side of the test tank. Hence, the focal individual could spend time in the zone near a predator or in the zone furthest away from a predator. In order to reduce disturbance from the outside, the experimental setups were placed in large oval tubs that were filled with aged tap water to the level inside the test tanks. The entire set-up was placed on a shelf of about 1 m height, and the observer was standing approximately 1.5 to 2 m away from the experimental setup and observed the fish diagonally from above.

To initiate a trial, we introduced the focal fish into the central tank and let it habituate to the test apparatus for 5 min. We then placed a predatory fish in either the right or the left auxiliary tank. The test tanks used in this study were relatively small, so focal fish were able to see the predator throughout the course of the experiment. Test fish would typically freeze on the bottom of the test tank for a few seconds (to some minutes) after the stimulus was introduced, so a trial began only after the focal fish had started to swim freely in the water column. We measured the time the focal fish spent in each preference zone during a 5-min observation period (*i.e.*, in the zone closest to or the zone furthest away from the predator). To detect side biases, the predator was switched between sides immediately after the first 5-min observation period and measurement was repeated.

In Treatment 1, focal fish were presented with a green swordtail (*Xiphophorus hellerii*) female (48.78 ± 1.25 mm standard length), which served as a control, since *X. hellerii* is a related, non-predatory species of similar body size, appearance and ecology to *P. mexicana*. In the second treatment, we presented focal individuals with *Cichlasoma salvini* (93.59 ± 3.16 mm) which is a native omnivorous cichlid in Southern Mexico and also includes mollies in its diet [37,52]. For the third treatment, we chose the algi- and detrivorous cichlid *Vieja bifasciata* (119.75 ± 1.97 mm) as a stimulus, and in Treatment 4, we presented a purely piscivorous predator, *Petenia splendida* (145.31 ± 3.19 mm) to focal individuals. All stimulus fish are common in natural *P. mexicana *habitats [37,38]. 

### 2.3. Statistical Analyses

We calculated an “avoidance score” as the dependent variable for the statistical analyses as: (time spent in the preference zone near the predator-time spent in the opposite preference zone). Scores were checked for assumptions of normality, homogeneity of variance and sphericity prior to conducting the statistical tests, which were performed using SPSS 13. All data are presented as mean ± S.E.M. (standard error of the mean); all graphical illustrations show estimated marginal means derived from the respective models outlined below.

#### 2.3.1. Experiment 1: Predator Recognition in Populations from Sulfide Springs

In our first experiment, we tested wild-caught *Poecilia *spp. from non-sulfidic and sulfidic habitats located in the Río Puyacatengo and Río Pichucalco drainages (see Table 1). While *P. mexicana* inhabits both non-sulfidic and sulfidic habitats in the Río Puyacatengo and non-sulfidic habitats in the Río Pichucalco, sulfidic springs along the Río Pichucalco are inhabited by the sulfur-endemic *P. sulphuraria*, a highly sulfide-adapted sister taxon to *P. mexicana *[31]. We compared patterns of predator avoidance using the avoidance score as dependent variable in a univariate General Linear Model (GLM) with drainage (Río Puyacatengo and Río Pichucalco), H_2_S (absent or present), predator type (four presented predators), sex (male or female), as well as all their interactions as independent variables. We included the predators' body size (standard length, SL) and the focal individuals’ body size (SL) as covariates. Interactions higher than the second order were removed from our final model because they were not statistically significant (*F* ≤ 1.35, *P* ≥ 0.37). We also removed the covariate “focal individuals” body size’ as it had no significant effect on avoidance scores (*F*_1, 188 _= 2.33, *P *= 0.13).

#### 2.3.2. Experiment 2: Predator Recognition in Cave-Dwelling *P. mexicana*

In our second experiment we compared predator avoidance reactions between individuals from a surface- and a cave-dwelling population in the Río Tacotalpa drainage. As representatives of a surface-dwelling population, we used fish from the sulfidic El Azufre, collected downstream the outflow of the sulfidic Cueva del Azufre. As representatives of a cave-adapted population, fish from cave Chamber II of the Cueva del Azufre were chosen for the experiment (Table 1; Figure 1). Predator avoidance scores were compared in a univariate GLM with predator type, sex, light regime (surface or cave), and their interactions as independent variables. Predators' body size and focal individuals' body size were included as covariates. We removed the non-significant third order interaction term (*F*_3,86_ = 0.22, *P *= 0.88) as well as focal individual body size (*F*_1,89_ = 1.01, *P *= 0.32) from the final model.

#### 2.3.3. Experiment 3: Influence of Predator Experience

In our third experiment we analyzed predator avoidance between predator-experienced (wild-caught) and naïve (laboratory-reared) individuals from the non-sulfidic Río Ixtapangajoya (Río Puyacatengo drainage). Avoidance scores were analyzed in a univariate GLM with predator type, sex, predator experience (laboratory-reared or wild-caught), and their interactions as independent variables. We also included predators' body size and focal individuals' body size as covariates but removed focal individuals' body size from the final model as it had no significant effect (*F*_1,96 _= 1.35, *P *= 0.25). Furthermore, we excluded all interactions from our final model, as none were statistically significant (*F* ≤ 2.00; *P* ≥ 0.12).

## 3. Results

### 3.1. Reduced Predator Avoidance in H_2_S-Adapted Poecilia?

**Figure 2:**
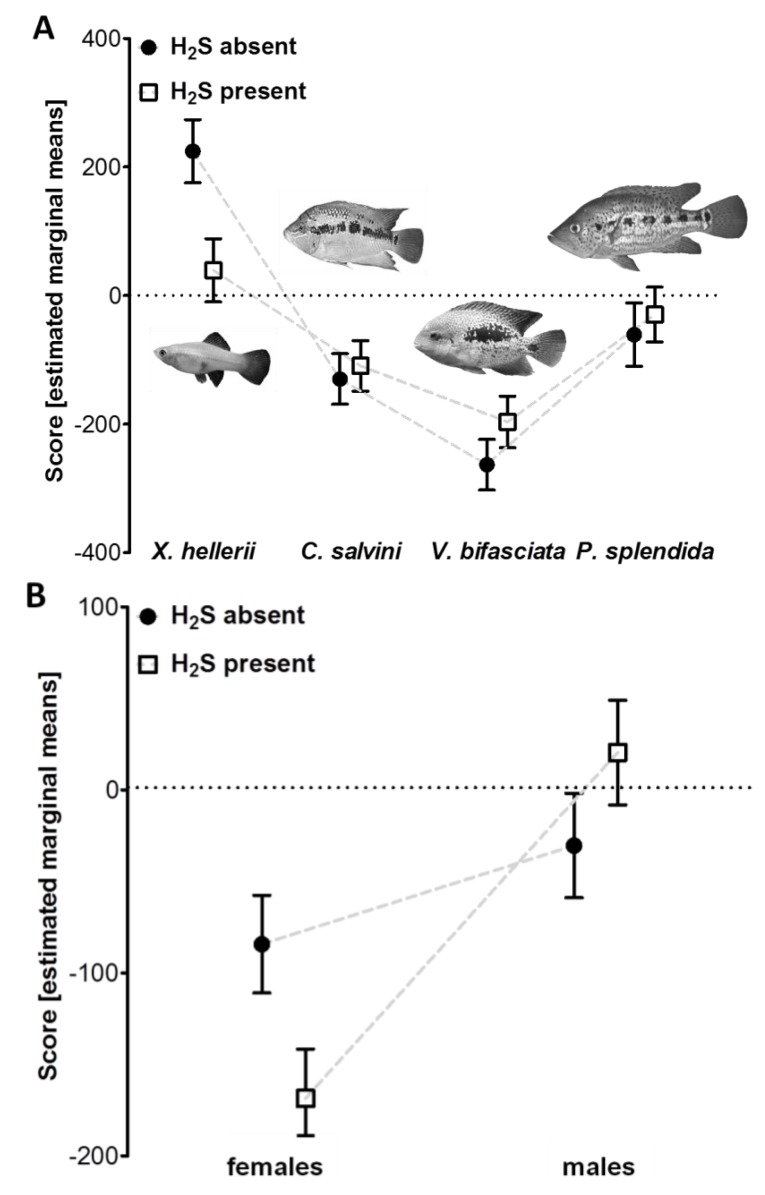
**(A)** Avoidance reactions of *Poecilia* spp. from sulfidic and non-sulfidic waters towards four different stimuli fish species. **(B)** Avoidance reactions of males and females adapted to either sulfidic or non-sulfidic waters. Positive values indicate that focal fish spent more time in the proximity of a stimulus while negative values indicate that focal fish avoided the proximity of a stimulus. Depicted are estimated marginal means (± S.E.M) of the avoidance score.

In our first experiment we compared patterns of predator avoidance between populations living either in non-sulfidic or sulfidic water from two different drainages. In our GLM, “predator type” was highly significant (Table 2A), indicating that test fish showed different responses towards the four stimulus species; more specifically, test fish showed avoidance behavior when confronted with the cichlids while they associated with swordtail females (Figure 2A). “Drainage” and “sex” were also significant, suggesting overall differences in the responsiveness between the Río Puyacatengo and Río Pichucalco drainage as well as between males and females (Table 2A).

**Table 2:** Results from General Linear Models (GLMs) with avoidance scores as the dependent variable. (**A**) Experiment 1; comparison between *Poecilia* spp. from sulfidic and non-sulfidic waters. (**B**) Experiment 2; comparison between surface and cave mollies. (**C**) Experiment 3; comparison between predator-experienced and naïve surface-dwelling *P. mexicana*. Significant effects are in boldface.

	*df*	*F*	*P*	partial eta^2^
**(A) Experiment 1**				
**predator type**	**3**	**21.72**	**<0.001**	**0.256**
**drainage**	**1**	**9.89**	**0.002**	**0.050**
H_2_S	1	0.36	0.55	0.002
**sex**	**1**	**19.28**	**<0.001**	**0.093**
**predator body size (SL)**	**1**	**16.00**	**<0.001**	**0.078**
predator type× drainage	3	1.92	0.13	0.030
**predator type ** **× H_2_S**	**3**	**4.25**	**0.006**	**0.063**
predator type× sex	3	1.30	0.28	0.020
drainage× H_2_S	1	0.61	0.44	0.003
drainage× sex	1	2.13	0.15	0.011
**H_2_S ** **× sex**	**1**	**5.95**	**0.016**	**0.030**
error	189			
**(B) Experiment 2**				
**predator type**	**3**	**14.43**	**<0.001**	**0.325**
sex	1	0.61	0.44	0.007
light regime	1	0.84	0.36	0.009
**predator body size (SL)**	**1**	**9.01**	**0.003**	**0.091**
predator type × sex	3	0.90	0.44	0.029
**predator type ** **× light regime**	**3**	**3.20**	**0.027**	**0.096**
sex × light regime	1	3.33	0.071	0.036
error	90			
**(C) Experiment 3**				
**predator type**	**3**	**16.01**	**<0.001**	**0.331**
sex	1	0.05	0.82	0.001
experience	1	0.01	0.91	<0.001
**predator body size (SL)**	**1**	**12.93**	**0.001**	**0.118**
error	97			

Most importantly, the interaction term “predator type × H_2_S” was significant (Table 2A), which suggests that fish from non-sulfidic and sulfidic habitats reacted differently towards the stimulus fishes (Figure 2A). Populations from sulfidic habitats showed a weaker predator avoidance reaction, but the strongest difference compared to populations from non-sulfidic habitats was found in a weaker association tendency with the swordtail females in the control treatment (Figure 2A). Also the interaction term of “sex × H_2_S” was found to have a significant effect (Table 2A) due to a stronger difference between the overall reaction of males and females from sulfidic habitats compared to a less pronounced difference between males and females from non-sulfidic waters (Figure 2B). The covariate “predator body size” had a significant effect (Table 2A) and we found a significant negative correlation between this covariate and standardized residuals obtained from our final model (Pearson correlation; *r*_p_ = −0.159, *P* = 0.022). We, thus, present all avoidance scores as estimated marginal means derived from our final model, which are corrected for predators' body size.

Overall, “predator type” had the strongest effect in the GLM (partial eta^2^ = 0.26), while the other two factors (“sex” and “drainage”) and all significant interactions were only of minor importance (partial eta^2^ < 0.09; Table 2A).

### 3.2. Reduced Predator Avoidance in Cave Mollies?

**Figure 3:**
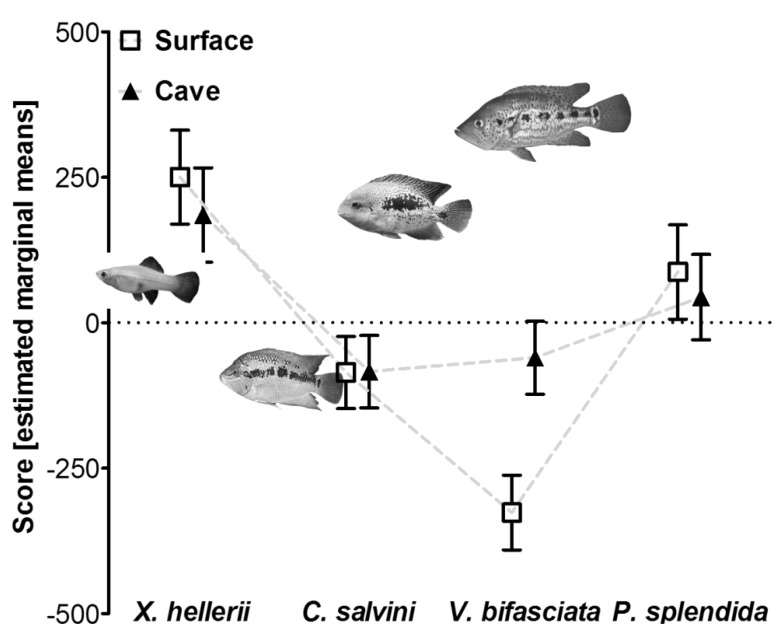
Avoidance reactions of surface- and cave-dwelling *P. mexicana* towards four different stimulus fish species. Positive values indicate that focal fish spent more time in the proximity of a stimulus while negative values indicate that focal fish avoided the proximity of a stimulus. Depicted are estimated marginal means (± S.E.M) of the avoidance score.

Our GLM analyzing predator avoidance scores of surface and cave-dwelling mollies from the Río Tacotalpa drainage revealed a significant effect of the factor “predator type” (Table 2B) indicating a general difference in the reaction towards the four predators presented in our experiment. The factors “sex” and “light regime” had no overall effect (Table 2B), and neither did the interaction terms “predator type × sex” and “sex × light regime” (Table 2B). Nevertheless, the interaction term “predator type × light regime” was significant (Table 2B), which was mainly due to the strong difference between the reaction of surface and cave-dwelling fish towards *V. bifasciata* (Figure 3). The covariate “predator body size” had a significant effect (Table 2B), and we found a significant negative correlation between this covariate and standardized residuals obtained from our final model (*r*_p_ = −0.15, *P* = 0.013).

### 3.3. Influence of Predator Experience on Predator Avoidance Responses in P. mexicana

**Figure 4:**
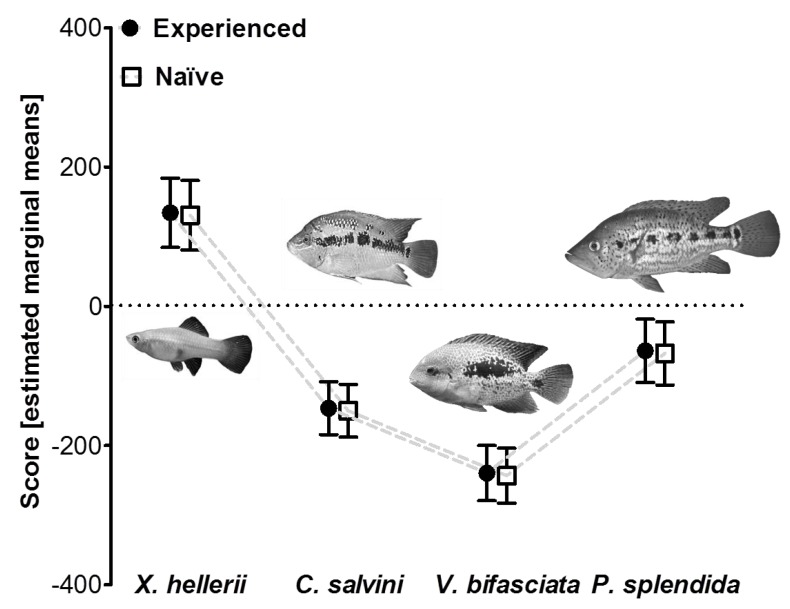
Avoidance reactions of predator-naïve (laboratory-reared) and predator-experienced (wild-caught) *P. mexicana* towards four different stimulus fish species. Positive values indicate that focal fish spent more time in the proximity of a stimulus while negative values indicate that focal fish avoided the proximity of a stimulus. Depicted are estimated marginal means (± S.E.M) of the avoidance score.

The GLM comparing avoidance scores of predator-naïve and predator-experienced surface mollies detected a significant effect of the main factor “predator type” (Table 2C; Figure 4) suggesting a general difference in individuals’ responses to the different predators used in our study. Both other main factors as well as their interactions had no significant effect (Table 2C) indicating that neither naïve and experienced fish nor males and females differed in their general responsiveness to the predator treatments. The covariate “predator body size” had a significant effect (Table 2C) and we found a significant negative correlation between this covariate and standardized residuals obtained from our final model (*r*_p_ = -0.207, *P* = 0.03).

## 4. Discussion

Our study investigated predator avoidance reactions in extremophile *Poecilia* spp. that either inhabit sulfidic springs or a sulfidic cave in southern México. In addition, we also investigated experiential effects on predator avoidance by comparing laboratory-reared and wild-caught fish. We found that *Poecilia* spp., regardless of origin or level of experience, avoided the proximity of cichlid fishes but associated with the poeciliid *X. hellerii*. The avoidance reaction towards cichlid stimuli correlated negatively with the body size of the stimulus fish, *i.e.*, larger cichlids elicited stronger responses. The results confirm our prediction that mollies from non-sulfidic waters avoid the proximity of the predatory cichlids *C. salvini* and *P. splendida.* Surprisingly, reactions were comparably strong or even stronger towards the non-predatory cichlid *V. bifasciata.* However, reactions towards non-predatory swordtail (*X. hellerii*) females were less consistent: Fish from sulfidic habitats in our first experiment showed a reduced tendency to associate with swordtails, while fish from a sulfidic spring in another drainage (Río Tacotalpa) did not show such a pattern in our second experiment. Furthermore, our results revealed that sex differences in the avoidance reactions are more pronounced in *Poecilia* spp. from sulfidic habitats, with females showing a stronger avoidance response than males. The comparison of predator-naïve (laboratory-reared) and predator-experienced (wild-caught) *P. mexicana *in our third experiment did not reveal any difference in predator avoidance reactions.

We initially proposed that energy limitation in concert with reduced piscine predation rates might favor the rapid reduction of costly anti-predator behaviors in extremophile *Poecilia *spp. populations. However, our results showed that avoidance reactions of fish from sulfidic and non-sulfidic habitats were only slightly reduced. This could be a sign of “ghost of predators past” mechanisms [12,53], assuming that not enough time has elapsed since the colonization of predator-devoid habitats to loose recognition mechanisms. Molecular phylogenetic evidence suggests that the sulfur creeks in southern Mexico were independently invaded by ancestral forms of *P. mexicana/P. sulphuraria‑* like fish not adapted to life in sulfidic waters [31]. Nonetheless, as outlined above, fish in all of these extreme habitats are probably energy-limited, which in turn, should impose strong selection on the reduction of costly and dispensable behaviors, like predator avoidance. Under these circumstances, the “ghost of the predators past” hypothesis seems unlikely. While we cannot exclude that anti-predator behaviors are pleiotropically linked to other behavioral traits that need to be maintained regardless of the presence or absence of predators [12,13], it is also possible that mollies adapted to sulfidic habitats at least occasional encounter some cichlid predators, as shoals of the predatory cichlid *C. salvini* can be observed in the mixing zones of non-sulfidic and sulfidic waters [18,19]. Still, most parts of the sulfidic springs are essentially devoid of predatory fishes, so we are inclined to argue in favor of another hypothesis—the “multi-predator hypothesis” assumes the presence of any type of predator to be sufficient to maintain anti-predator behaviors—even for missing predators [9]. Riesch *et al.* [25] found *P. mexicana* from sulfidic habitats to experience 20-fold increased avian predation rates. This increased predation pressure by birds could promote the persistence of avoidance reactions towards predatory fishes.

The strongest difference between mollies from sulfidic habitats and those from non-sulfidic habitats in our first experiment was a reduced tendency of mollies from sulfidic habitats to associate with swordtail females. In all sulfidic habitats, we regularly observe enormous shoals of mollies (often more than 1000 individuals) performing ASR [23,24]. This raises the question why mollies from those habitats showed a reduced tendency to associate with a non-predatory, similar-sized fish, which would be interpretable as a reduced shoaling tendency. In the Trinidadian guppy (*Poecilia reticulata*) it was found that an association preference for swordtails could be induced when juveniles were raised together, and imprinting was suggested as a possible mechanism [54]. No preference was found when guppies had not had the ability for social learning, *i.e.*, when both species were not raised together [54]. In our case, this explanation seems to be rather unlikely though, as swordtails are absent from the sulfidic springs and only occasionally found in the less toxic mixing zones [19,20]. Still, cave mollies and surface mollies from the sulfidic creek (El Azufre) in Experiment 2, as well as laboratory-reared surface mollies in Experiment 3—all of which had no prior experience with swordtails—did show the same high tendency to associate with *X. hellerii *females. However, *Poecilia* spp. in Experiment 1 did associate with the swordtail females at least to some degree, which is in stark contrast to the avoidance of any cichlid stimuli, demonstrating that focal fish were able to evaluate the swordtail as not threatening. We do not have a compelling explanation for this pattern at hand but suggest that shoaling behavior (*i.e.*, the tendency to associate with other fish evaluated as not representing a threat) might be reduced in sulfide-dwelling fish inhabiting the Ríos Puyacatengo and Pichucalco. Why this is so, and why fish from the sulfidic El Azufre do not show such a reduction in shoaling requires further experimentation in the future.

We also found more pronounced sex differences in predator avoidance in mollies from sulfidic habitats, and we suggest population- and sex-specific differences in boldness to account for this pattern: Riesch *et al.* [20] found females from sulfidic habitats to be more cautious than their counterparts from non-sulfidic habitats, while males from both non-sulfidic and sulfidic waters—overall bolder than females—did not differ in their mean boldness. In the Trinidadian guppy, boldness traits differ in relation to the ambient predation regime, with females from habitats with low-predation risk (due to the lack of cichlid predators) being more cautious compared to females from high predation habitats, while males indifferently are bolder [55]. In line with these findings, our current results hint towards piscine predation as the driving force in the evolution of population- and sex-specific variation of boldness in our system.

In our second experiment we asked whether surface and cave forms of *P. mexicana* differed in their level of predator avoidance. Apart from a major difference in the reaction to *V. bifasciata*, surface- and cave-dwelling fish showed very similar responses to the different stimuli, comparable to those observed in fish from non-sulfidic surface habitats in Experiments 1 and 3 (*i.e.*, predator avoidance in response to the other cichlids and shoaling in response to swordtail females). This is contrary to our prediction that cave fish, which live in an energy-limited environment devoid of piscivorous fishes, should rapidly lose their predator avoidance responses through regressive evolution or a lack of predator-experience. However, the Cueva del Azufre, though lacking piscine predators, is not entirely predator-free [33,34], and as outlined for our first experiment, we cannot properly exclude any of the three hypotheses, but argue that the “multi-predator hypothesis” [9] most likely explains the persistence of anti-predator responses in cave fish.

Interestingly, both surface and cave fish showed no reduced tendency to associate with swordtail females, which was predicted for cave mollies based on the results of previous studies testing for species discrimination of *P. mexicana* females when given the choice between a conspecific and a swordtail stimulus female [56] and generally reduced shoaling behavior in cave mollies [39]. Indeed, shoaling in the absence of piscine predators (as in the Cueva del Azufre) loses its benefits but still comprises costs related to intraspecific competition [57]. As the size of the stimulus shoal in the previous study (four conspecific fish [40]) implies higher intraspecific costs than a single swordtail female in our current study, we tentatively suggest that our experimental design may not be appropriate to detect differences in shoaling behavior.

Several studies on teleost fish investigated how different anti-predator behaviors are shaped by experience (*P. reticulata *[2]; *P. mexicana *[18]; *Gobiusculus flavescens* [3]). However, results are not congruent, and the relative importance of experience in shaping anti-predator behavior remains unclear. In our third experiment, we, therefore, asked if there are differences in predator avoidance between laboratory-reared and wild-caught individuals, *i.e.*, between predator-naïve and predator-experienced fish. We could not detect significant differences between groups, which leads us to assume that mere predator avoidance (moving away from a predator), as measured in our experimental design, could possibly be the simplest form of anti-predator behavior not shaped through experience. For example, predator-naïve two-spotted gobies (*G. flavescens*) appear to react strongly to the presentation of any kind of big fish, but are incapable of recognizing predator-specific odors, unless they experienced them in conjunction with a threat cue [3]. Botham *et al.* [58] found avoidance reactions in guppies to be shaped by experience only in populations that evolved in predator-rich environments. Our experiment uncovered no effect of learning on the predator avoidance behavior of fish from a predator-rich (non-sulfidic) site, but the question of whether the learning component of predator avoidance differs between ecotypes of *Poecilia* spp. remains to be studied in detail.

Surprisingly, focal fish in all three experiments (except cave mollies) showed the strongest avoidance when presented with the non-predatory cichlid *V. bifasciata*. We qualitatively observed that individuals of *V. bifasciata* were very active throughout the trials while the two other cichlid species remained rather calm when transferred into the test tank. We, thus, hypothesize that focal fish might have perceived *V. bifasciata* as more threatening, and the observed stronger responses could be an artifact of our test design. Furthermore, a study on coral reef fishes [59] showed that predatory fish share common morphological features like broad heads and big mouths that are used by prey fishes as cues to recognize predators. We hypothesize that the typical “cichlid-shape” of *V. bifasciata* leads the test fish to anticipate a predator and, in combination with the higher activity levels, elicits a strong response. We propose the use of predator models (*i.e.*, video animations of calmly swimming predators) in future studies to standardize the testing procedure (e.g., to exclude the effects of activity differences) and to analyze common features (in body shape or coloration) of predators that might elicit avoidance responses.

Our present study concentrated on visual cues in predator recognition, while future studies will have to elucidate a potential role of alarm pheromone-based predator recognition in extremophile *Poecilia *spp., as chemical cues have been shown to play an important role for predator recognition in poeciliid fishes (see [60,61]). However, it remains unclear what effect the presence of H_2_S would have on such chemically mediated cues. For example, studies in the poeciliid genus *Xiphophorus* suggest that disruption of pheromone-based female choice may be caused by (anthropogenic) water pollution [62]. Testing for the responses to “alarm cues” may provide a fruitful opportunity to test an alternative hypothesis explaining—at least in part—our present results: The lack of a difference in the response to (predatory) cichlids between fish from benign and extreme habitats could be explained by our focus on visual cues, as visual presentation of a potential predator represents an extreme risk to the prey fish. In this context one can hypothesize that the response towards chemical cues (representing lower predation risk) could indeed be reduced in extremophile fish.

## 5. Conclusions

In summary, *Poecilia* spp. living in extreme habitats with impoverished piscine predator regimes did not show reduced predator avoidance responses when presented with predatory cichlids that are found in regular non-sulfidic habitats. Nevertheless, extreme habitats are not predator-free environments, as either birds and/or invertebrates prey on extremophile mollies. Thus, we hypothesize that predator avoidance is still functional in line with the “multi-predator hypothesis” which assumes the presence of *any* kind of predator to be sufficient to maintain anti-predator behavior. A general avoidance of cichlids can be ascribed to cichlid-specific morphological features shared by non-predatory and predatory cichlid species. It remains to be determined what causes the observed variance in the tendency to associate with the non-predatory poeciliid *X. hellerii*. Differences in the response to different predator species cannot be explained through learnt predator recognition, as predator-naïve and predator-experienced fish did not differ in their responses. Our current study, therefore, highlights the importance of inherited avoidance mechanisms towards piscine predators, which are not reduced even when populations invade energy-limited environments largely devoid of predatory fishes.
